# Intrusive growth of primary and secondary phloem fibres in hemp stem determines fibre-bundle formation and structure

**DOI:** 10.1093/aobpla/plv061

**Published:** 2015-05-27

**Authors:** Anastasia Snegireva, Tatyana Chernova, Marina Ageeva, Simcha Lev-Yadun, Tatyana Gorshkova

**Affiliations:** 1 Kazan Institute of Biochemistry and Biophysics, Kazan Scientific Centre, Russian Academy of Sciences, Kazan 420111, Russia; 2 Department of Biology and Environment, Faculty of Natural Sciences, University of Haifa - Oranim, Tivon 36006, Israel

**Keywords:** Cambium, *Cannabis sativa*, hemp, intrusive growth, phloem fibre bundles, plant fibres

## Abstract

Plant fibres—cells with important mechanical functions and a widely used raw material—are usually identified in microscopic sections only after reaching a significant length or after developing a thickened cell wall. We characterized the early developmental stages of hemp (*Cannabis sativa*) stem phloem fibres, both primary (originating from the procambium) and secondary (originating in the cambium), when they still had only a primary cell wall. We gave a major emphasis to the role of intrusive elongation, the specific type of plant cell growth by which fibres commonly attain large cell length. We could identify primary phloem fibres at a distance of only 1.2–1.5 mm from the shoot apical meristem when they grew symplastically with the surrounding tissues. Half a millimeter further downwards along the stem, fibres began their intrusive elongation, which led to a sharp increase in fibre numbers visible within the stem cross-sections. The intrusive elongation of primary phloem fibres was completed within the several distal centimetres of the growing stem, before the onset of their secondary cell wall formation. The formation of secondary phloem fibres started long after the beginning of secondary xylem formation. Our data indicate that only a small portion of the fusiform cambial initials (<10 %) give rise directly or via their derivatives to secondary phloem fibres. The key determinant of final bundle structure, both for primary and secondary phloem fibres, is intrusive growth. Through bi-directional elongation, fibres join other fibres initiated individually in other stem levels, thus forming the bundles. Our results provide the specific developmental basis for further biochemical and molecular-genetic studies of phloem fibre development in hemp, but may be applied to many other species.

## Abstract (precis)

Plant fibres – cells with important mechanical functions and a widely used raw material – are usually identified in microscopic sections only after reaching a significant length or after developing a thickened cell wall. We characterized the early developmental stages of hemp stem phloem fibres, both primary and secondary, when they still had only a primary cell wall. We gave a major emphasis to the role of intrusive elongation, the specific type of plant cell growth, by which fibres commonly attain large cell length. Intrusive growth is the key determinant of final bundle structure, both for primary and secondary phloem fibres.

## Introduction

A fibre is an individual plant cell belonging to the sclerenchyma. Its major characteristics are: (i) a very significant length (from several hundreds of micrometres up to many centimetres) with the ratio between cell length and diameter ranging from 50 to 2000 or even more; (ii) an extraordinarily thick cell wall, reaching up to 15 µm; and (iii) mechanical function as the major one (Esau 1977; Fahn 1990; van Dam and Gorshkova 2003; Gorshkova *et al*. 2012). Plant phloem fibres, including those of hemp (*Cannabis sativa*), have been used by humans since ancient times and currently have numerous applications, both traditional and modern (Reichert 1994; Ebskamp 2002; Thygesen *et al*. 2006; John and Thomas 2008). The long and strong hemp fibres have a rich history of use for the production of ropes, paper and textiles. At present, hemp fibres are successfully used in innovative technologies, e.g. as reinforcement in composite materials (Thygesen *et al*. 2006; Abot *et al*. 2013). Besides practical importance, plant phloem fibres are interesting for fundamental studies of plant cell growth and cell wall formation, exhibiting a special (intrusive) type of elongation (Esau 1977; Lev-Yadun 2001; Snegireva *et al*. 2010; Gorshkova *et al*. 2012), and in various fibre types a special (gelatinous) tertiary cell wall that can serve as ‘plant muscles’ and pull upward trunks and branches by fibre-cell shortening (Mellerowicz *et al*. 2008; Gorshkova *et al*. 2010, 2012; Mikshina *et al*. 2013).

At early stages of development primary fibres grow symplastically (Erickson 1986) with the surrounding tissues, without separation of the adjacent cell through middle lamellae; later, fibres start to grow intrusively (Esau 1977; Fahn 1990; Gorshkova *et al*. 2012). The development of fibres was considered for a long time to combine cell-tip intrusive growth, ongoing even for months, simultaneously with secondary cell wall thickening of the ‘older’ central fibre part (Anderson 1927; Esau 1977; Fahn 1990). However, recently it was established that in flax (*Linum usitatissimum*), the elongation of primary phloem fibres is separated in time from secondary cell wall thickening (Gorshkova *et al*. 2003, 2005). The question remains, whether this pattern of cell wall thickening in flax fibres is the rule or just a special case. Is the same true for different plant species and for secondary phloem fibres (produced by the cambium, rather than the procambium as in flax)?

Hemp and flax are phylogenetically distant species; according to Angiosperm Phylogeny Group III (AGP 2009) they belong to different orders of angiosperm plants—Rosales and Malpighiales, respectively. Similar to flax, hemp stems produce primary phloem fibres, which initiate close to the shoot apical meristem (SAM); but unlike flax, they also produce secondary phloem fibres, which originate from the vascular cambium (Magitt 1948; Crônier *et al*. 2005; De Pauw *et al*. 2007).

In general, hemp stem anatomy is well known (Magitt 1948; Bonatti *et al*. 2004; Crônier *et al*. 2005; Blake *et al*. 2008) and is typical for dicotyledonous plants with secondary stem thickening, provided by the activity of the vascular cambium. The cross-sectional shape of the mature hemp stem, which can reach a height of 3 m, changes from nearly circular at the base to ribbed in the middle and in the upper parts; the number of ribs in upper stem parts is usually eight (Ordina 1978). The ribs are rich in collenchyma and primary phloem fibres. Lower stem internodes contain several rings of secondary phloem fibre bundles (Chernova *et al*. 2005; Blake *et al*. 2008).

Both primary and secondary phloem fibres, being components of the primary and secondary phloem, respectively, are the extra-xylary bast fibres that form bundles like the bast fibres in many other plant species. Fibre-bundle shape (round, square, flat, etc.) and density (the number of bundles per unit area) may vary, as well as the number of fibres within a specific bundle, which typically ranges from 10 to 40 as seen in the bundle's cross-section (McDougall *et al*. 1993). However, there are no detailed data on the developmental processes leading to bundle formation.

The development of hemp fibres has been studied with advanced techniques such as cDNA microarrays (De Pauw *et al*. 2007; van den Broeck *et al*. 2008). However, the basic developmental fibre biology, a pre-requisite for interpreting experimental results and for effectively planning and performing further experiments, is largely unknown, especially for the early stages of fibre development. As in other plant species, hemp fibres are usually identified only when they are quite long or after they have developed thickened cell walls. The earliest stages of, hemp fibre development including bundle formation have received very little, if any attention.

Here we studied the early stages of development of primary and secondary phloem fibres in hemp stems, with special attention to the formation of phloem fibre bundles. This was done in comparison to the better known primary phloem flax fibres (Ageeva *et al*. 2005; Snegireva *et al*. 2010). We found that the fibre elongation processes are basically similar in these two species and that intrusive growth is a major determinant of the final fibre bundle's structure, for both primary (hemp and flax) and secondary (hemp) phloem fibres.

## Methods

### Plant material

The monoecious cannabinoids-free green-stem hemp cultivars Diana and Ingreda were grown on experimental fields of the Chuvash Research Institute of Agriculture (Tsivilsk, Russia, 55°52′N, 47°29′E, sod-podzolic soil, natural daylight and watering) according to regional agricultural standards. The developmental data were similar for both Diana and Ingreda cultivars; therefore, we present only those from the analysis of cultivar Diana stems.

**Figure 1.**
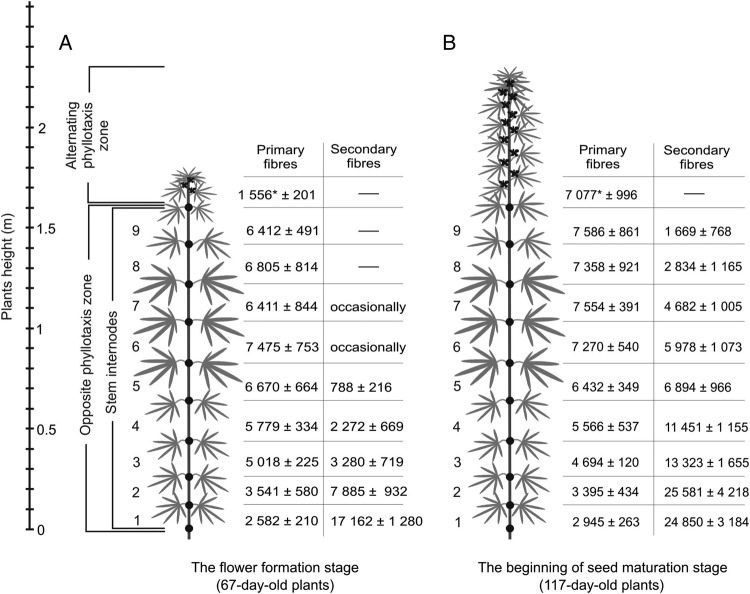
Scheme of plant sample collection and number of primary and secondary phloem fibres in the cross-sections of hemp stems in various internodes in (A) 67-day-old and (B) 117-day-old plants. To the left, stem height (m) and 1 to 9—number of a stem internode. In the upper part of the stem with alternating phyllotaxis, fibres were counted at a distance 10 mm from the SAM for 67-day-old plants, which corresponds to the middle of the upper part of 117-day-old plants. *Significant difference determined by Student's *t*-test (*P* < 0.05) in number of primary phloem fibres in 67- and 117-day-old plants. Other than in the top part of the stem there are no statistically significant changes in primary phloem fibre numbers in any stem internode through the 50-day interval. Thus, the intrusive elongation of the primary phloem fibre is completed within the top part of growing hemp stem.

Plants for the analysis of stem cross-sections were sampled at two developmental stages: at the time of flower formation (67-day-old plants), and in the beginning of seed maturation (117-day-old plants). The height of plants chosen for analysis at the flower formation stage was 172 ± 5 cm, with the upper 24 ± 5 cm having alternating phyllotaxis, while in the lower stem parts the phyllotaxis was opposite (Fig. 1). At the beginning of the seed maturation stage the plants selected for analysis were 229 ± 11 cm tall and the alternating phyllotaxis zone increased to ∼65 ± 21 cm. The plants sampled for analysis at both stages had nine internodes with opposite phyllotaxis, numbered from the bottom of the stem.

### Histology, cell counts and measurements

For identification of primary phloem fibres at early stages of development, samples of the apical part of hemp stem (upper 0.5 cm) were fixed according to a standard procedure in 2.5 % (v/v) glutaraldehyde in 0.1 M phosphate buffer (pH 7.2) for 4 h, rinsed and post-fixed for 2 h in 1 % (w/v) OsO_4_. After dehydratation in an ethanol series, acetone and propylene oxide, the samples were embedded in Epon resin PELCO^®^ (Ted Pella, Redding, CA, USA). Sections (3 µm thick) were cut on an ultramicrotome LKB 8800 (LKB, Mariehäll, Sweden), and stained with 1 % toluidine blue for 10–20 s. Samples (0.5 cm) at 1 cm from the shoot apex and more basal were cut free hand and stained with 0.5 % toluidine blue in water or 0.003 % Calcofluor White (Megazyme, Wicklow, Ireland) in Tris-buffer for 3 min.

Serial transverse sections for investigation of fibre thickening dynamics were made from the stems of 67-day-old plants. To analyze the early stages of secondary cell wall formation in primary phloem fibres, 5-mm long tissue samples were taken from the upper stem part (6 cm from the shoot apex), and for secondary fibres from the sixth internode from the stem's base, in which cell wall thickening of secondary phloem fibres had just began. Samples were fixed and embedded by the same method as described above. Sections (3 µm thick) were cut with 9–12 μm steps on an ultramicrotome LKB 8800, and stained with 1 % toluidine blue. Fibre tips of nine primary and seven secondary phloem fibres were found and followed on serial sections through 150 μm of tissue.

The length of cambial initials of hemp stems was determined in serial tangential sections. Samples from the eighth internode of 117-day-old plants were fixed and embedded by the same method as described above. Tangential longitudinal sections (5 µm thick) were cut on an ultramicrotome LKB 8800, and stained with 1 % toluidine blue. A total of 20 cambial initial cells from each of the five plants were measured (*n* = 100).

To count the number of fibres on the stem cross-sections, five plants of each of the two growth stages were sampled for the measurements. The samples were taken from the middle of each internode and fixed in 80 % ethanol. The number of fibres was counted on stained free-hand cut transverse sections within an one-eighth sector of the stem area. For that the cover glass was divided into eight sectors and its centre was aligned with the stem centre in the cross-sections.

The length of the primary and secondary phloem fibres was measured after isolation of fibres from 117-day-old plants. For that, fibre-rich peels from the third internode of ethanol fixed stems were incubated in 1.2 % Macerozyme (Serva, Heidelberg, Germany) in 100 mM phosphate buffered saline (PBS) (pH 7.0) overnight at 4 °C. After washing in the same buffer, samples were transferred to glass slides, where bundles of primary or secondary fibres were separated from the surrounding tissues with the aid of a needle. Individual fibres were isolated from the bundles and examined by light microscopy (×100) to ensure their integrity and individuality. Their length was measured under a stereomicroscope (Zeiss, Jena, Germany) at a magnification of ×12.5.

The fibre diameters, the number of secondary phloem fibre bundles and the number of fibres within a bundle were determined on free-hand cut cross-sections taken at the middle part of the several internodes (from 1 to 5) of four 117-day-old plants (*n* = 50).

Bundle structure of primary and secondary phloem fibres was examined in fibre-enriched peels of mature stems after fixing in 80 % ethanol and partial maceration (1.0 % Macerozyme, Serva, overnight) and clearing the samples by boiling for 1 min in concentrated lactic acid.

All tissue specimens, except for isolated fibres, were observed using an LSM 510 Meta confocal microscope (Zeiss) with UV excitation from a HBO mercury vapour lamp for Calcofluor White fluorescence, or using the transmitted light detector and argon-ion laser (stained with toluidine blue) and photographed with an AxioCam HRs camera (Zeiss).

Statistical analyses were done using Microsoft Excel. Values in tables are means ± standard errors.

## Results

### Identification of primary phloem fibres in early stages of development

**Figure 2.**
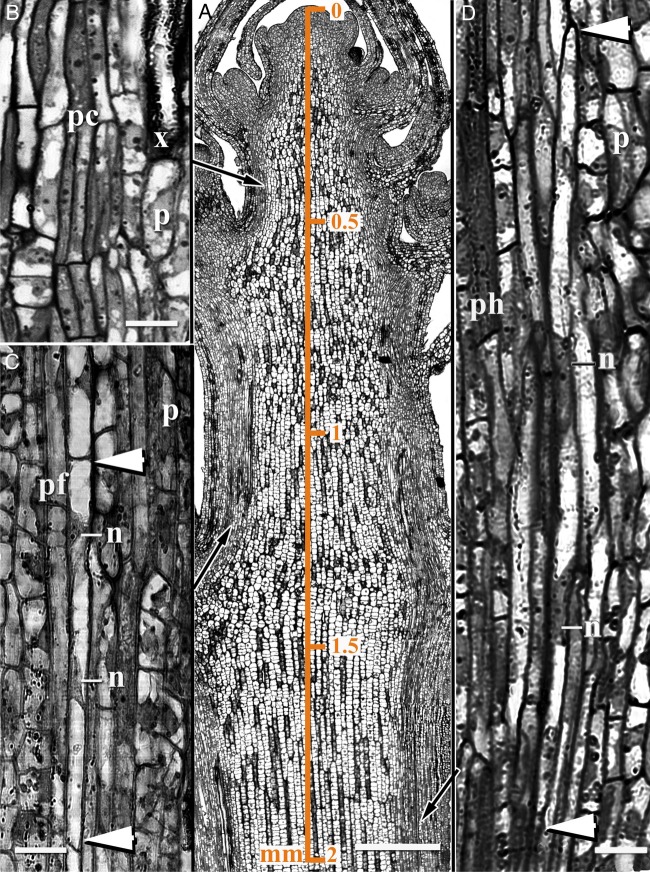
Early stages of primary phloem fibre development in hemp. (A) A longitudinal almost median section of the apical part of the hemp stem showing (B) procambial cells, (C) primary phloem fibres at the stage of symplastic growth and (D) primary phloem fibres at the stage of intrusive growth. Procambial strands that can be identified at 0.4 mm from the SAM give rise to primary phloem fibres (pf). At a distance of 1.2–1.5 mm from the SAM, parenchyma cells (p) are found between the procambium (pc) and the fibres, confirming that the procambium had ceased to form primary fibres below this stem region. Primary phloem fibres at the stage of symplastic growth—narrow, elongated cells with at least two elongated nuclei (n)—still have flat ends (marked by arrowheads). Fibre ends become tapered when the intrusive growth of primary fibres starts. Arrows indicate position of B, C and D on A. Distance (mm) from the shoot apex is indicated in A. n, nuclei; ph, phloem; x, xylem. Bar: A = 200 µm, B–D = 10 µm.

On longitudinal sections of the apical zone of developing hemp stems (Fig. 2A), procambial strands were identified at a distance of only 0.4 mm from the SAM (Fig. 2B). At 0.8 mm from the SAM, primary xylem vessel members as well as primary phloem sieve tubes were found. It was possible to identify individual young primary fibres, visible at the periphery of the primary phloem at a distance of only 1.2–1.5 mm from the SAM (Fig. 2C). In this region, differentiating fibres were narrow (3–4 µm in diameter) and elongated (70–200 µm) cells with at least two elongated nuclei. At this stage of development the fibres still had flat ends, indicating that the young fibres still elongated symplastically along with their neighbouring cells.

Initiation of intrusive primary fibre growth could be determined because of the characteristic ‘knees’, which arise when the flat ends of symplastically growing fibres change into the tapered ones of intrusively growing cells (e.g. Ageeva *et al*. 2005). For primary phloem hemp fibres such structures were identified at a distance of 1.8–2.0 mm from the SAM. Very soon, both fibre ends became tapered (Fig. 2D).

### Analysis of the intrusive elongation of primary phloem fibres on stem cross-sections

**Figure 3.**
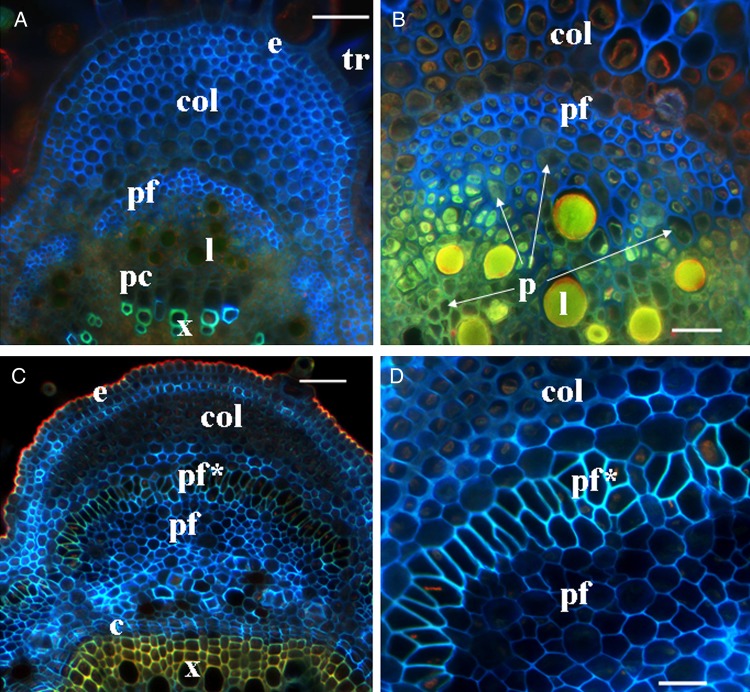
Cross-sections of the top part of a hemp stem, stained with Calcofluor White. (A and B) Primary phloem fibres at a distance of 10 mm from the SAM. The fibre bundles are being formed in the inner part of the cortex; parenchyma cells (p) and laticifers (l) are present between the fibres and the procambium. (C and D) Primary phloem fibres at a distance of 80 mm from the SAM. Bundles of primary phloem fibres (pf) are already formed meaning that the intrusive growth has ceased. Fibres located at the bundle side closest to the stem periphery are the first to start secondary cell wall deposition. e, epidermis; col, collenchyma; pc, procambium; pf*, primary phloem fibres with thickened cell wall; tr, trichome. Bar: A and C = 50 µm, B and D = 20 µm.

In the cross-sections of apical parts of hemp stems, fibres were easily detected at a distance of ∼10 mm from the SAM (Fig. 3A and B). Groups of fibres could be distinguished from the surrounding parenchyma cells by their characteristic angular cell shape and lighter cytoplasm (probably because of the large central vacuole). Fibre identification in the cross-sections was confirmed by studying longitudinal sections (showing their elongated shape) of the same stem part (data not shown). Primary phloem fibres formed large bundles within each stem rib and some smaller bundles in between the ribs, with an average of 16 ± 2 bundles in the whole cross-section. The bundles of primary phloem fibres were formed in the inner part of the cortex (Fig. 3A and B). Parenchyma cells and laticifers were found between the procambium and the fibres. The presence of other type cells between the procambium and the fibres confirmed that procambium had ceased to form primary fibres above this stem region. Within fibre bundles, parenchyma-type cells, but more elongated than typical cortical chlorenchyma cells, as seen in longitudinal sections, were present. The total number of primary phloem fibres in the stem cross-sections of 67-day-old plants sharply increased from 1556 ± 201 at 10 mm from the SAM up to 6118 ± 392 at a distance of 60 mm from the SAM (Table 1).

**Table 1.** Number of primary phloem fibres in hemp stem cross-sections taken at different distances from the SAM in 67-day-old plants. *Differences from the sample below in the table are significant, determined by Student's *t*-test (*P* < 0.05).

Distance from the SAM (mm)	Number of fibres
10	1556* ± 201
30	4672* ± 687
60	6118 ± 392
90	7232 ± 1103

Within the upper part of the stem, all primary phloem fibres seen in the cross-sections had only primary cell walls. Thin secondary cell walls could be detected in some primary fibres by light microscopy at a distance of ∼60 mm from the SAM. At the distance of 80 mm from the SAM, the fibres located at the bundle side closest to the stem periphery were the first to start secondary cell wall deposition (Fig. 3C and D). The distance from the SAM where all primary phloem fibres had started secondary cell wall deposition was ∼1000 mm, corresponding to the fifth internode from the bottom of the 67-day-old stem.

**Figure 4.**
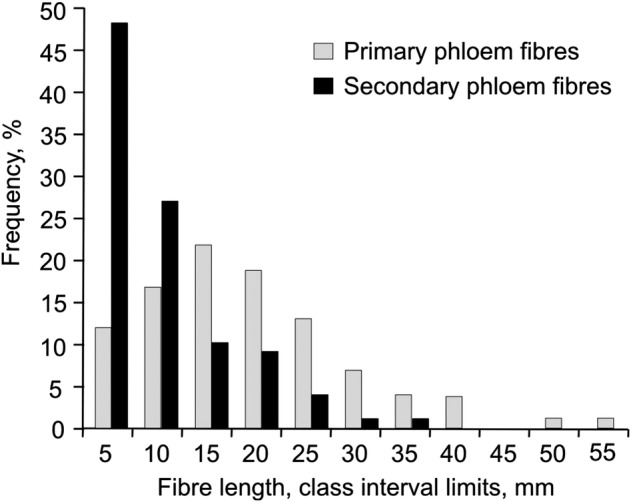
Distribution of primary (grey) and secondary (black) phloem fibre length in mature hemp stem (frequency of abundance of different length fibres). Primary phloem fibres are significantly longer than secondary ones. *n* = 100.

Intrusive growth of primary phloem fibres can be revealed by the comparison of cross-sections at the same stem level through different stages of plant development, since the intrusive fibre elongation leads to an increase in the number of fibres seen in the cross-section at a certain point with time (Gorshkova *et al*. 2003; Snegireva *et al*. 2006). The significant increase in the numbers of primary phloem fibres at a given cross-section position between the plants sampled at a 50-day interval was observed only in the upper stem part, which at the first sampling date (67-day-old plants) was still close to the SAM (Fig. 1). The number of primary phloem fibres in the cross-section at 10 mm from the SAM of 67-day-old plants (Table 1) was only ∼20 % of that found at the same stem level 50 days later (Fig. 1B), two-thirds at 30 mm from the SAM, but remained almost the same at 60 mm. More than 60 mm from the SAM there were no statistically significant changes in primary phloem fibre numbers through the 50-day interval in any stem internode (Fig. 1). Thus, the intrusive elongation of primary phloem fibres was completed within the upper 60 mm of the hemp stem. Considering the growth rate of hemp stems (2 cm per day), intrusive growth of primary phloem fibres lasts no more than 3 days. The final fibre length, mostly attained by intrusive growth, was determined after partial maceration of mature tissues and was on average 17.5 ± 1.6 mm, with a maximal length of 55 mm (Fig. 4).

### Development of secondary phloem fibres

**Figure 5.**
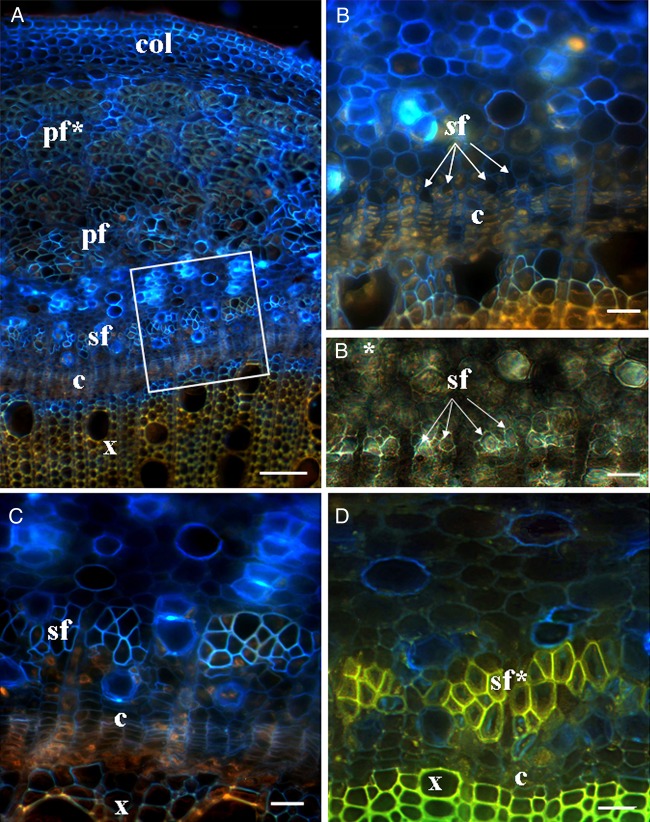
Cross-sections of a hemp stem, from the middle part of seventh internode stained with Calcofluor White. (A–C) Sections of 67-day-old plants. (A) A general view of the stem part; a white rectangle marks the area of secondary fibres and corresponds to C. (B) Stem area above A (B*—part of B in transmitted light channel); small groups of the intrusively growing secondary phloem fibres (marked by arrows) near the cambium. (C) Bundles of secondary phloem fibres with primary cell walls. (D) Secondary phloem fibres with thickened cell walls in 117-day-old plants at the same level as in B. The vascular cambium (c) in the hemp stems starts to produce secondary phloem fibres only at a distance of 600–700 mm from the SAM (seventh internode from the plant base). c, cambium; col, collenchyma; pf, primary phloem fibres; pf*, primary phloem fibres with thickened cell wall; sf, secondary phloem fibres; sf*, secondary phloem fibres with thickened cell wall. Bar: A = 100 µm, B–D = 20 µm.

Secondary phloem fibres are formed by the vascular cambium or its immediate derivatives. In hemp stems, the vascular cambium starts to produce the secondary xylem at a distance of ∼80 mm from the SAM (Fig. 3C), but secondary phloem fibres could be detected only much lower along the stem, at a distance of 600–700 mm from the SAM (in the seventh internode from the plant base) (Fig. 5A). The onset of secondary phloem fibre formation varied between individual hemp plants, and sometimes fibres could be detected only in the sixth internode from the plant base (Fig. 1). On the stem cross-sections, small groups of cells near the cambium characterized by a dark cell lumen could be distinguished from other cell types and their size and location corresponded to those of the secondary phloem fibres (Fig. 5B). At this stage the fibres had only primary cell wall clearly seen in transmitted light (Fig. 5B*). In older stem parts, the number of cells in such groups increased gradually, reaching the number of fibres in mature secondary fibre bundles; cell wall thickness was similar in all fibres in a bundle (Fig. 5C). Fibre identification in the cross-sections was confirmed by studying longitudinal sections (that showed their elongated shape) of the same stem part (data not shown). In 117-day-old plants, bundles of secondary phloem fibres with thickened cell walls were found in this stem area (Fig. 5D).

In the cross-sections of the lower hemp stem internodes, the number of secondary phloem fibres ranged from a few thousand to tens of thousands at any given stem height and significantly increased through the 50-day interval (from 67 to 117-day-old plants) (Fig. 1). Within the same plant, the number of secondary phloem fibres found in stem cross-sections was the largest at the plant bottom, while the number of primary phloem fibres was smaller in the oldest, basal internodes (Fig. 1).

The length of axial cambial initials of hemp stems, determined in tangential longitudinal sections, was 0.254 ± 0.051 mm (*n* = 100). Secondary phloem fibres have no stage of symplastic elongation, because they are formed from the cambium in non-elongating stem regions. Therefore, secondary phloem fibres attain their final lengths only via intrusive growth. The average length of secondary phloem fibres isolated from mature plants was 7.6 ± 0.7 mm (Fig. 4). Thus, the pronounced intrusive growth of secondary phloem fibres led to an increase of ∼30-fold in their cell length. The diameter of secondary phloem fibres was smaller than that of the primary ones (7.9 ± 0.6 μm versus 29.5 ± 1.3 μm, respectively).

**Figure 6.**
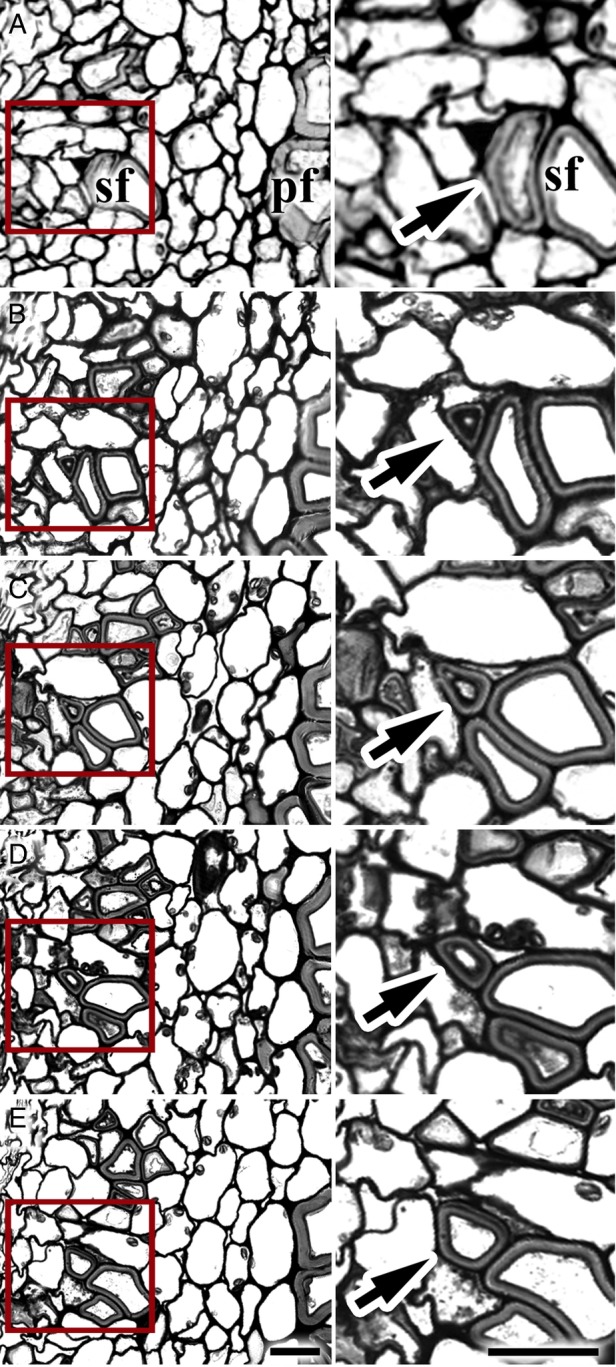
Serial cross-sections of a stem in the region of secondary phloem fibres, showing the cell wall thickness in the tip and middle part of one and the same fibre. Right column—larger magnification of the area marked on the left by a red rectangle. In the enlarged area shown in the right panel the fibre tip is indicated by an arrow. Phloem fibres have quite uniform thickness of the cell wall throughout their cell length. Therefore, fibre elongation and cell wall thickening do not coincide. pf, primary phloem fibres; sf, secondary phloem fibres. Distance between: A and B = 20 µm, B and C = 50 µm, C and D = 100 µm, D and E = 150 µm. Bar = 20 μm.

The serial cross-sections of both secondary (Fig. 6) and primary (data not shown) phloem fibres taken at the beginning of secondary cell wall deposition revealed the quite uniform thickness of the cell wall throughout the whole cell length. A situation of a phloem fibre having a thickened cell wall in its central part and only a primary cell wall at the fibre tip was never observed in our material.

**Figure 7.**
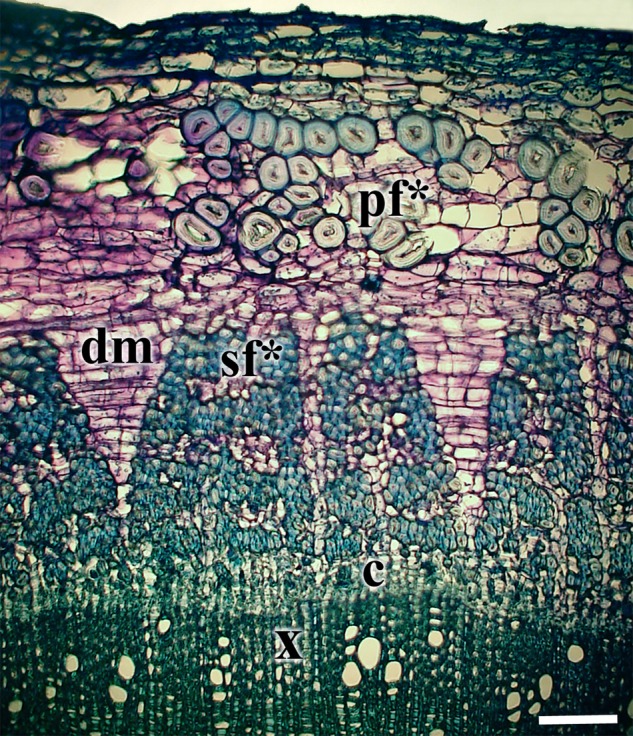
Cross-section of a hemp stem from the middle part of the first internode of a 117-day-old plant stained with toluidine blue. Note the formation of dilatation meristems (dm) in two rays between the secondary phloem fibre bundles and the difference in diameters of primary and secondary phloem fibres. c, cambium; pf*, primary phloem fibres with thickened cell wall; sf*, secondary fibres with thickened cell wall; x, xylem. Bar = 50 µm.

In hemp, the stem's cambium gives rise to secondary phloem fibres alternating with other cell types of the secondary phloem. Such variable differentiation of cambial derivates results in concentric rings of bundles of secondary phloem fibres. In our study, the maximal number of such alternating fibre-bundle rings was four (Fig. 7). The number of secondary phloem fibres within a bundle was on average 22 ± 7. The secondary phloem fibres with only primary cell walls were observed only in the innermost secondary phloem fibre ring, closest to the cambium, and never detected in the second or outer secondary phloem fibre rings. The bundles of secondary phloem fibres appeared in the cross-sections to be more compact than those of primary phloem fibres.

**Figure 8.**
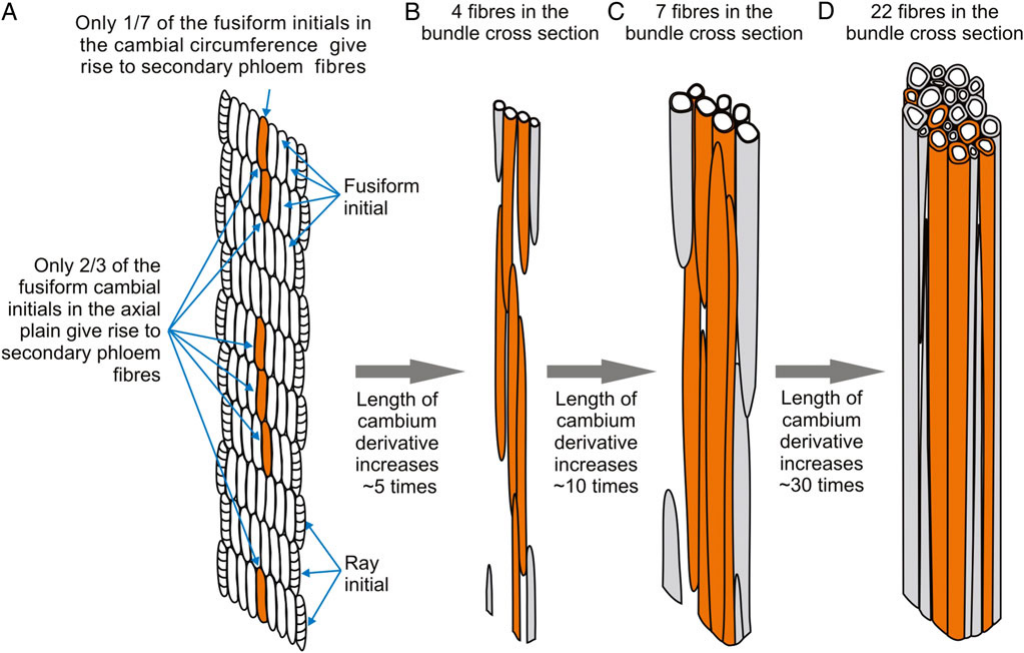
A scheme of the development of the secondary phloem fibre bundles demonstrating the major role of intrusive fibre growth. (A) Cambium region giving rise to secondary phloem elements (including secondary phloem fibres). Only a small portion of the fusiform cambial initials (one-seventh of the cambium circumference and two-third of the axial plane; marked in orange) give rise directly or via their derivatives to secondary phloem fibres. (B–D) Sequential stages of secondary fibre-bundle formation. Fibres in the bundle, which were formed by cambial initials located above and below that axial plane, are given in grey. Due to considerable increase in length by bi-directional intrusive elongation, fibres join other fibres initiated individually in other stem levels, thus forming the bundles and leading to the increase of fibre numbers on the cross-section. (D) Mature secondary phloem fibre bundle (fibres ceased intrusive growth and thickened cell wall).

The average increase in the number of secondary phloem fibres (20- to 30-fold), as seen from the fibre numbers found in a bundle in cross-sections is smaller than the average increase in fibre length (30-fold), arrived by comparing the length of cambial initials and the length of mature secondary phloem fibres. From that it can be concluded that the fusiform cambial initials, which give rise to secondary phloem fibres, do not form continuous axial polar lines of fibres just above or below each other, but rather are axially separated by one or two fusiform cambial initials that on average do not give rise to fibres (Fig. 8). The possibility that this is a general developmental rule cannot be concluded without a broad comparative study of many taxa.

The structure of the vascular cambium in the hemp stem can thus be deduced by examination of secondary tissue patterns as seen in the stem cross-section (e.g. Larson 1994 and citations therein). The spacing of the unicellular rays of hemp secondary xylem indicated that the cambial ray initials in hemp are laterally separated within the cambium by 7 ± 2 fusiform initial files on average. Each radial secondary phloem block contains one fibre bundle belonging to a certain bundle ring and in accordance with the above discussion on intrusive fibre growth, the cambial sector that forms this block has only a single fusiform cambial initial that gives rise to a secondary phloem fibre. Keeping in mind that (i) only about one-seventh of the fusiform initials in the cambial circumference and (ii) only about two-third of the fusiform cambial initials in the axial plain give rise to secondary phloem fibres, it can be stated that only a very small portion of hemp cambial cells, probably <10 %, give rise to secondary phloem fibres.

In the lowest internodes, the stems were considerably thickened (12.5 ± 2.4 mm in diameter, as compared with 1.5 ± 0.7 mm at 3 cm from the SAM). In thicker stem parts, the distances between fibre bundles in the outer secondary fibre-bundle rings, sometimes increased due to dilatation growth following cell divisions and expansion of ray parenchyma (Fig. 7).

**Figure 9.**
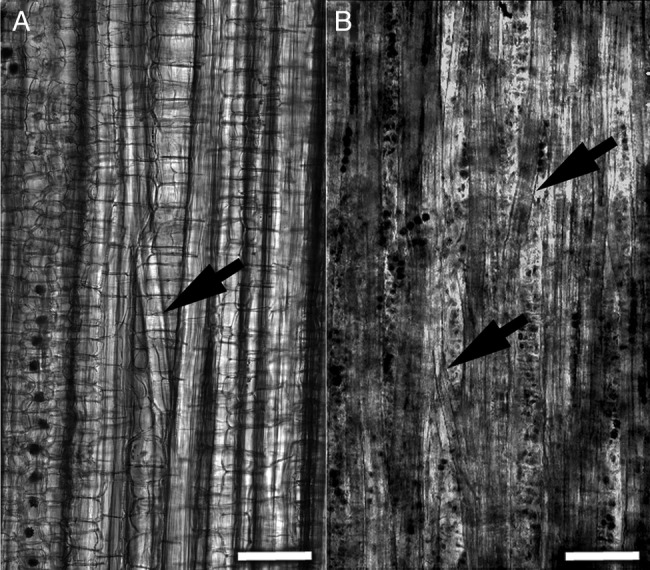
Structure of the (A) primary and (B) secondary phloem fibre bundles on the strips peeled off from the third internode of a 117-day-old plant hemp stem. Bundles frequently split and merge along the stem forming numerous anastomoses (marked by arrows). Bar = 100 µm.

In the longitudinal peels of stem barks, many secondary phloem fibre-bundle anastomoses were found, leading to the formation of a fibre-bundle network (Fig. 9B). Some anastomoses also occurred among the bundles of the primary phloem fibres (Fig. 9A).

**Figure 10.**
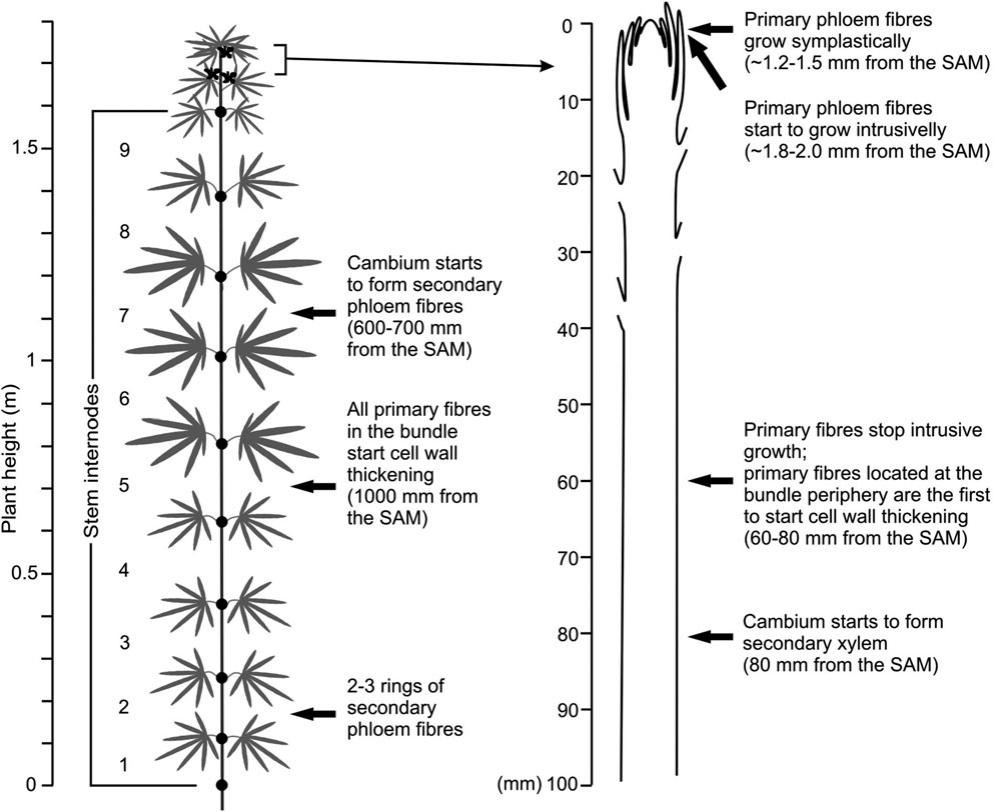
Scheme of the location of primary and secondary phloem fibres at different developmental stages within the stem of a 67-day-old hemp plant. For details, see text.

The summary of the results is presented in Fig. 10.

## Discussion

### Location of the major stages of phloem fibre development in hemp stems

A single hemp plant produces 700 000–800 000 long primary phloem fibres and ∼2 million—shorter secondary ones (Chernova *et al*. 2005). Primary phloem fibres can be identified at a distance of only 1.2–1.5 mm from the SAM. At this stage they still grow symplastically with the surrounding tissues. Half a millimeter further downwards along the stem, the primary phloem fibres begin their intrusive elongation, much before the cessation of symplastic growth of the surrounding tissues, as suggested by the continued increase in the distance between successive leaves in these internodes (Fig. 2A). In the upper 5–6 cm of the stem, the intrusive growth is very pronounced so that the number of primary phloem fibres seen in the cross-sections sharply increases long after the procambium can give rise to new primary phloem fibres, and at a distance of only 3 cm from the SAM reaches 60 % of the final number (Table 1). The intrusive elongation of primary phloem fibres ends within the top 6 cm of growing hemp stems, as evident by reaching the maximal fibre number per bundle and per stem cross-section at that distance from the SAM. Therefore, biochemical and molecular studies of intrusive growth of primary phloem fibres should be conducted in this stem section, corresponding to up to 3 days of growth. The fact that the fibre bundles are in the process of formation very close to the SAM, led many who sampled more mature internodes to think that they are formed by the primary meristem as bundles and to overlook the process of their formation by the gradual association of fibres to bundles via intrusive growth. This misunderstanding has resulted from erroneous conduction and interpretation of various experiments on the role of hormones in primary phloem fibre formation, resulting in the confusion between GA-induced fibre initiation and GA-induced fibre elongation and bundle formation (e.g. Aloni 1979).

In the upper 600–700 mm of the stem, corresponding to 25–30 days of axial growth, the growing hemp stem is still devoid of secondary phloem fibres. Their formation starts long after the development of a vascular cambium ring and the onset of secondary xylem differentiation. The exact duration of secondary phloem fibre intrusive growth is rather difficult to elucidate. There is considerable variation in where the cambium begins to form secondary phloem fibres along the stem in different hemp plants. This makes the statistical evaluation of secondary fibre number at early stages of development less precise than that of primary phloem fibres. However, the existence of secondary phloem fibres without thickened cell walls only in the fibre bundles that are the closest to the cambium suggests that their intrusive elongation does not last more than a couple of weeks, as is also indicated by the increase in the number of secondary phloem fibre rings through the 50-day period (i.e. from two to four in the first internode).

### Phloem fibre elongation and cell wall thickening do not coincide

It was believed for decades that while plant fibres grow by intrusive apical elongation they deposit secondary cell walls in the older, central parts of the cell (Esau 1977; Fahn 1990). However, in the top stem part, intrusively elongating primary phloem fibres have only primary cell walls. Thickening of cell walls in primary hemp phloem fibres occurs only further away from the SAM, indicating that in primary phloem fibres, at the cellular level, cell elongation and thickening of cell walls are two separate processes. To prove that the stages of cell elongation and cell wall thickening of primary and secondary hemp phloem fibres are separate, we studied serial stem cross-sections (Fig. 6). The examination of these serial cross-sections revealed that along individual primary and secondary phloem fibres, cell wall thickness was quite uniform, including the tip. Thus, hemp phloem fibres do not form secondary cell walls while elongating, a character that is probably a rule since it is also true for flax (Gorshkova *et al*. 2003) and *Arabidopsis thaliana* (Zhong *et al*. 2001). The fact that both primary and secondary phloem fibres do not thicken cell walls while they elongate does not mean that those stem parts are incapable of producing secondary cell walls. In both the young upper stem parts and older, more basal parts, vessel members form thick secondary cell walls (Esau 1977; Fahn 1990). This is a very strong developmental indication for cell-type-specific regulatory mechanisms of cell wall formation, and their molecular and genetic features should be studied while taking this cell-type specificity into account. Indeed, two different types of the NAC family of transcription factors, NST and VND act specifically as master regulators for the lignification of fibres (Zhong *et al*. 2008, 2010, 2011; Gorshkova *et al*. 2012) and vessel members (Kubo *et al*. 2005; Zhong *et al*. 2010; Gorshkova *et al*. 2012), respectively. We posit that for the non-lignified thick cell walls of gelatinous fibres the involvement of other or additional regulatory genes is expected.

### Intrusive growth is the major determinant of phloem fibre-bundle formation

Examination of cross-sections of mature hemp stems and stems of many other taxa shows many phloem fibre bundles. This may give the wrong impression that the fibre bundles are formed as such. However, the study through the various stages of fibre-bundle ontogeny we conducted revealed a very different situation. Intrusive growth, the special type of growth occurring when the rate of a cell elongation exceeds that of neighbouring cells or tissues (Esau 1977; Lev-Yadun 2001; Snegireva *et al*. 2010; Gorshkova *et al*. 2012), rather than the initiation of many fibres at a certain position of the procambium or cambium, leads to the gradual increase of fibre-cell numbers in individual bundles (Gorshkova *et al*. 2003; Snegireva *et al*. 2006). For instance, the numbers of primary phloem fibres in stem cross-sections increases significantly only in the upper 6 cm of the growing stem (Table 1, Fig. 1). The increase in fibre numbers in this stem segment is due only to intrusive growth, since fibre initiation from the temporary promeristem (the procambium) ends very close to the SAM, since at distance of only 10 mm from the SAM parenchyma cells and laticifers were well developed in between the procambium and the primary fibres (Fig. 3A). Thus, the formation of bundles of primary phloem fibres is mostly based on intrusive elongation.

In the beginning of the intrusive growth stage, cell length in both the primary and the secondary phloem fibres is of the same range: ∼200 μm for primary fibres at the end of symplastic growth and ∼254 μm for cambial initials that give rise to secondary fibres. Similarly to the data of others (Magitt 1948; Sankari 2000), we have determined that mature primary phloem fibres are several times longer than the secondary ones (Fig. 4). Thus, the intrusive growth of primary phloem fibres is more pronounced. However, even for the secondary phloem fibres the intrusive growth is still quite impressive, leading on average to a 30-fold increase in cell length. It means that the bundles of secondary phloem fibres that typically contain 20–30 cells at any specific stem height are formed as a result of intrusive elongation of fibres initiated both above and below the studied section plane (Fig. 8). It follows that for a certain cross-section position of a certain fibre bundle, the cambium does not produce a group of fibres, but rather only a single fibre and the formation of secondary phloem fibre bundles is totally based on the significant intrusive elongation of individual fibres.

### Comparison of the early stages of phloem fibre development in hemp and flax

The anatomy of the hemp stem is more complicated than that of flax since several additional primary and secondary tissues, such as collenchyma, laticifers and secondary phloem fibres exist in hemp. Stem thickness is commonly only ∼1.5–2.0 mm in the upper stem parts even in mature flax plants and not much more even in the thicker lower parts, while in the analysed hemp stems it reached this thickness already in the apical part of the plant and increased significantly downwards along the stem. The ‘snap point’—the region of the stem (usually 6–8 cm from the SAM), where a sharp increase in stem mechanical strength protects it from breaking when pulled manually—serves in flax as a good indicator of primary phloem fibre's developmental transition from intrusive growth to cell wall thickening (Gorshkova *et al*. 2003). In hemp, the ‘snap point’ is not so well pronounced. The absence of a well-pronounced ‘snap point’ in hemp may be due to the presence of a large amount of collenchyma, which in the absence of primary phloem fibres with thickened cell walls serves a mechanical function and strongly supports the developing young stem. All the above-mentioned differences make the developmental and following this, the molecular analyses of hemp stems, much more complicated than in flax.

The results of this study allow us to compare the general similarities and differences in the formation of phloem fibres in two phylogenetically distinct classic higher plant fibre crop species such as hemp and flax. The major similarities in the early stages of development of the primary phloem flax fibres (Gorshkova *et al*. 2003; Ageeva *et al*. 2005; Snegireva *et al*. 2010) with those of hemp, and of both the primary and secondary phloem fibres that originate from two different meristems (the procambium and the vascular cambium) within the hemp plants studied here, include the following. (i) Primary phloem fibres in both plant species start to elongate by symplastic tissue elongation. (ii) The onset of intrusive elongation of primary phloem fibres is coupled with the formation of a ‘knee’, when the flat ends of symplastically growing fibre cells are transformed into tapered ends of intrusively elongating ones. (iii) Intrusive elongation of primary phloem fibres occurs only in the upper several centimetres of the stem. (iv) Intrusive elongation is never combined with cell wall thickening within an individual fibre cell, as was proposed earlier (Esau 1977; Fahn 1990), but rather it continues for a limited time and is completed before the onset of cell wall thickening. (v) The primary phloem fibres located in the periphery of the bundles, facing the epidermis, are the first to start cell wall thickening, in accordance with the optimality of least material investment and maximal mechanical strength (e.g. Wainwright *et al*. 1976). Fibres in the inner part of a bundle may remain with only primary cell walls for a longer time (in 67-day-old hemp stems they can be found up to almost a metre below the SAM). However, they do not elongate at this stage as the number of fibres seen in bundle cross-sections does not change any more. (vi) During fibre elongation, primary phloem fibres become multinuclear (Snegireva *et al*. 2010). (vii) The extent of intrusive fibre elongation largely determines the size (measured in fibre numbers as seen in a cross-section) of bundles of phloem fibres. The longer the individual fibres are, the more opportunities they have to join other fibres and generate larger and denser bundles (Fig. 9). Short fibres simply cannot reach and join fibres located in other internodes or even in further parts of the same internode.

The major differences between primary phloem fibres of flax and hemp are in the structure of their bundles. In hemp, parenchyma cells are present within primary phloem fibre bundles, even in the young bundles (Fig. 3) and the primary phloem fibre bundles frequently split and merge along the stem forming numerous anastomoses (Fig. 9A). In flax, the primary phloem fibre bundles are very compact, never containing parenchyma cells (Gorshkova *et al*. 2003) and there are no anastomoses between the bundles. Therefore, on longitudinal bark peels of flax, its primary phloem fibre bundles look like long ‘columns’, but bundles of primary and secondary phloem fibres in hemp form ramified anastomizing networks. Some anastomoses exist already at early stages of primary phloem fibre-bundle development as evident by the parenchyma cells that are present within the young bundles (Fig. 3). However, anastomoses may get even more pronounced when hemp stem structure is modified during the intensive secondary stem thickening and the phloem fibre bundles as seen in the cross- and longitudinal sections get more separated due to dilatation of the ray parenchyma via cell divisions and cell expansion (Fig. 7).

Secondary phloem fibres of hemp stems differ from the primary fibres of both flax and hemp, in the following aspects. (i) They originate from a different meristem—the vascular cambium rather than the procambium. (ii) They have only a single nucleus rather than many (Snegireva *et al*. 2010). (iii) Their final cell length and diameter are considerably smaller than that of the primary fibres (Fig. 4; Magitt 1948; Sankari 2000).

Thus, our results show the many basic similarities in the development of phloem fibres between flax and hemp, indicating that many characteristics of the better known bast fibres of flax may indeed represent a common developmental and structural situation. The approaches presented here to study phloem fibre-bundle formation can be used to analyze the development of phloem fibres of other taxa in comparative studies. Our results also provide the developmental basis, including both the positional data and the time frame for further developmental, biochemical and molecular-genetic studies of various stages of phloem fibre development in hemp and probably other taxa as well.

## Sources of Funding

This work was partially supported by the grant from Russian Foundation for Basic Research (grant number 15-04-05721).

## Contributions by the Authors

All authors jointly conceptualized the project and experimental strategy. A.S., T.C. and M.A. performed the experiments. All authors analysed the data and wrote the manuscript.

## Conflict of Interest Statement

None declared.
